# A Case of Primary Hyperparathyroidism With a Hypercalcemic Crisis Resulting in Distinct Bone Mineral Redistribution

**DOI:** 10.7759/cureus.72430

**Published:** 2024-10-26

**Authors:** Kazuhiro Nomura

**Affiliations:** 1 Department of Nutrition and Metabolism, Tokushima University Graduate School, Tokushima, JPN; 2 Center for Diabetes and Endocrinology, The Tazuke Kofukai Foundation Medical Research Institute Kitano Hospital, Osaka, JPN

**Keywords:** bone mineral redistribution, calcium, hypercalcemia, parathyroid hormone, primary hyperparathyroidism

## Abstract

Primary hyperparathyroidism (PHPT) is generally detected early, but this case involves a rare hypercalcemic crisis associated with a parathyroid adenoma. A 66-year-old man presented with extreme fatigue and loss of appetite. Serum calcium and parathyroid hormone (PTH) levels were elevated to 22.5 mg/dL and 3100 pg/mL, respectively. After the initial management of hypercalcemia, parathyroidectomy confirmed a benign adenoma. This case demonstrates a notable redistribution of bone minerals, with a significant decrease in cortical bone density but preservation, and even enhancement, of trabecular bone density. This redistribution highlights the complex dual action of PTH on bone metabolism, emphasizing the need for careful monitoring in severe PHPT cases.

## Introduction

Primary hyperparathyroidism (PHPT) is characterized by excessive secretion of parathyroid hormone (PTH), which disrupts calcium homeostasis and profoundly affects bone metabolism. Advances in diagnostic tools have enabled earlier detection of PHPT, often during asymptomatic stages [1-3]. However, life-threatening hypercalcemic crises, while rare, still occur [4,5]. This case highlights such a crisis, demonstrating distinct patterns of bone mineral redistribution, likely induced by extreme and prolonged elevations of PTH.

## Case presentation

In December 2019, a 66-year-old man was admitted to our hospital with severe fatigue and appetite loss. Three days prior, he had experienced increasing thirst and dizziness and accidentally fell asleep while driving, resulting in a traffic accident. The patient had no significant medical history, and there were no notable conditions in his family history. Upon admission, he measured 159 cm in height and weighed 58 kg. His blood pressure was elevated at 186/96 mmHg with a regular pulse of 80 beats per minute. Physical examination revealed an elastic soft mass on the left neck, approximately 3 cm in size, without erythema or edema. Laboratory data upon admission are summarized in Table 1. Notably, plasma calcium levels were elevated to 22.5 mg/dL, accompanied by high urinary calcium excretion (614.4 mg/day) and elevated intact parathyroid hormone (PTH) level (3100 pg/mL). Additionally, levels of 1,25-dihydroxyvitamin D and osteocalcin were elevated while alkaline phosphatase (ALP) and PTH-related protein (PTHrP) were within the normal range (Table 1). Bone mineral density (BMD) was significantly reduced at the distal radius (T score: right; -2.76, left; -2.12) but paradoxically increased at the L2-4 spine (T score: +1.31).

**Table 1:** Laboratory data on admission AST: aspartate aminotransferase; ALT: alanine transaminase; LDH: lactate dehydrogenase; ALP: alkaline phosphatase; γ-GTP: gamma-glutamyl transpeptidase; BUN: blood urea nitrogen; PTH: parathyroid hormone, PTHrP: PTH-related protein, 1,25-(OH)2D: 1,25-dihydroxyvitamin D

CBC		Normal range	
WBC	14,100	3,500-9,000	/μl
RBC	470x10^4^	450-650x10^4^	/μl
Platelet	20.7x10^4 ^	15.0-40.0x10^4^	/μl
Blood chemistry			
AST	49	10-40	IU/l
ALT	63	5-40	IU/l
LDH	650	124-222	IU/l
ALP	112	38-113	IU/l
γ-GTP	101	<70	IU/l
Alb	4.5	3.8-5.2	g/dl
Glu	99	70-109	mg/dl
BUN	39.2	8.0-22.0	mg/dl
Cre	1.85	0.61-1.04	mg/dl
Na	143	136-147	mEq/l
K	4.4	3.6-5.0	mEq/l
Cl	105	98-109	mEq/l
Ca	22.5	8.8-10.4	mg/dl
P	5.1	2.4-4.3	mg/dl
Mg	1.9	1.8-2.6	mg/dl
Endocrine examination			
Intact-PTH	3100	10-65	pg/ml
PTHrP	<=1.0	<=1.0	pmol/l
1,25-(OH)_2_D	98.1	20.0-60.0	pg/ml
Osteocalcin	34.4	8.4-33.1	ng/ml
Urine chemistry			
Ca	614.4	100-300	mg/day
P	492.8	500-2,000	mg/day
Cre	1.27	1.0-1.5	g/day
Ccr	38	90-120	ml/min

**Figure 1:**
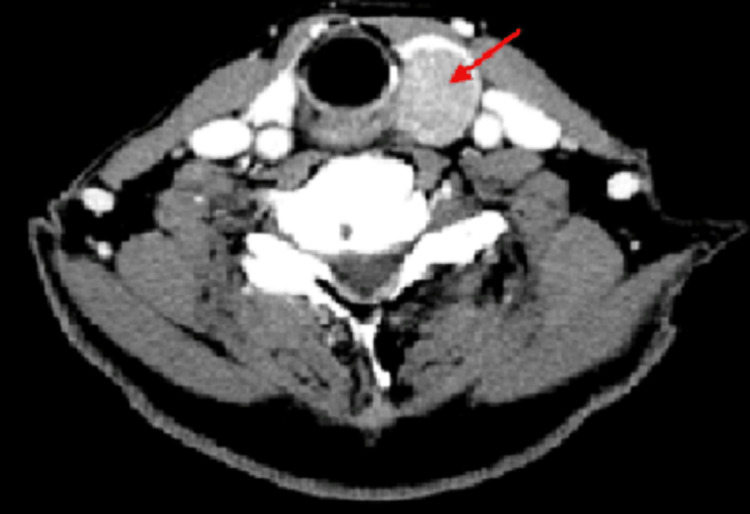
CT examination CT of the neck showed a tumor located on the posterior aspect of the left thyroid lobe. Tumor indicated by the red arrow.

**Figure 2:**
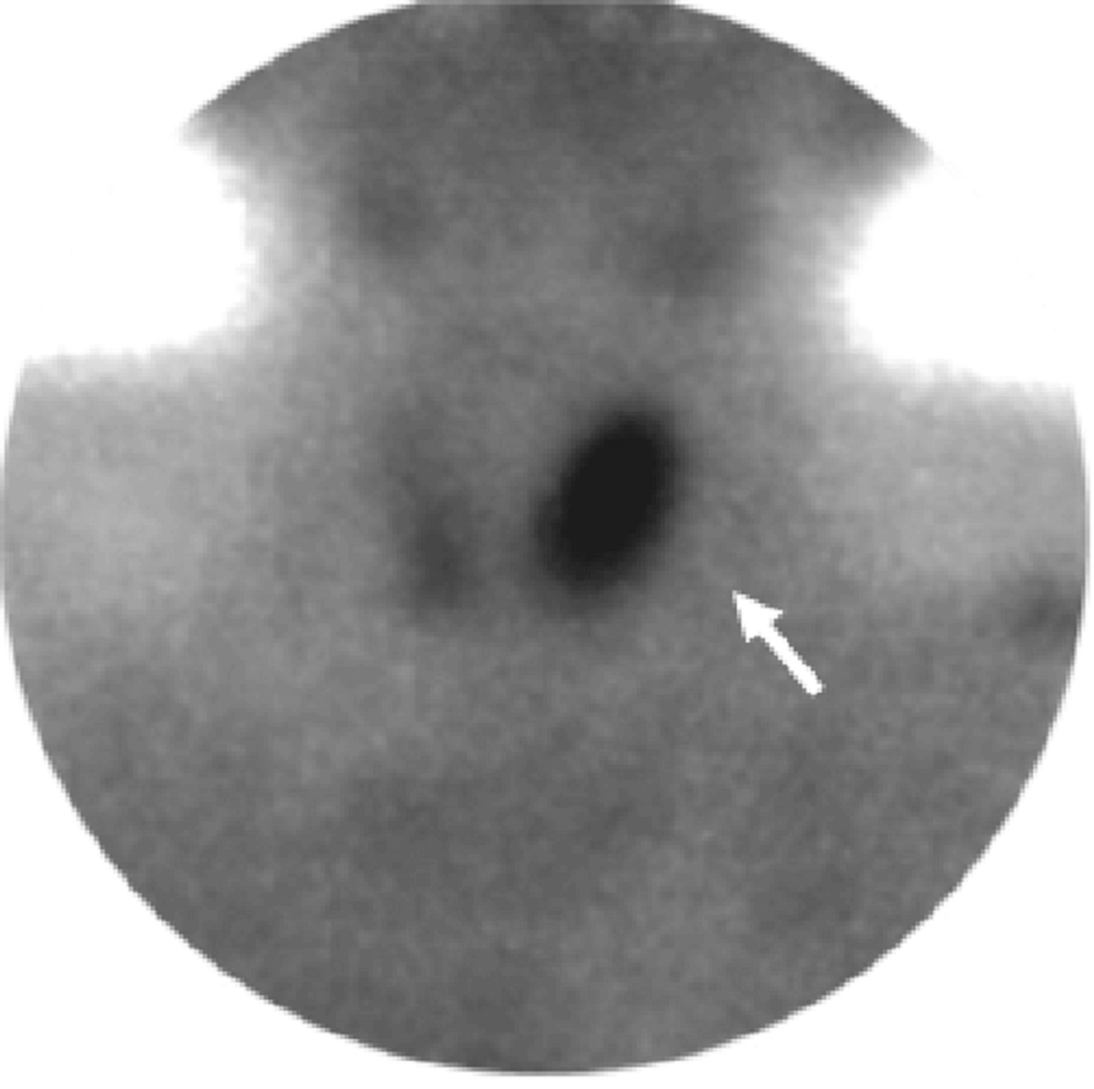
99mTecnecium methoxy-isobutyl-isonitrile (MIBI) scintigraphy 99mTecnecium MIBI scintigraphy demonstrated uptake in the same location on the left thyroid lobe. Tumor indicated by the white arrow.

Computed tomography (CT) confirmed a 24 x 18 mm tumor in the same location (Figure 1). Additionally, 99mTecnecium methoxy-isobutyl-isonitrile (MIBI) scintigraphy showed uptake in the same region (Figure 2). Other imaging studies, including magnetic resonance imaging (MRI) of the head and CT of the chest and abdomen, were unremarkable.

Based on these findings, a diagnosis of primary hyperparathyroidism was made, with concerns regarding potential parathyroid carcinoma. Given the severity of hypercalcemia, immediate measures were taken to reduce serum calcium levels while further diagnostic studies were conducted. Hydration was initially managed with massive saline infusion but serum calcium levels remained above 20 mg/dL. Hemodialysis was subsequently employed, lowering serum calcium to around 15 mg/dL. Intravenous bisphosphonates (pamidronate disodium 30 mg/week, administered three times), loop diuretics (furosemide 40 mg/day), and calcitonin (80 u/day intramuscularly) were also used, successfully reducing calcium level to 12 mg/dL.

**Figure 3:**
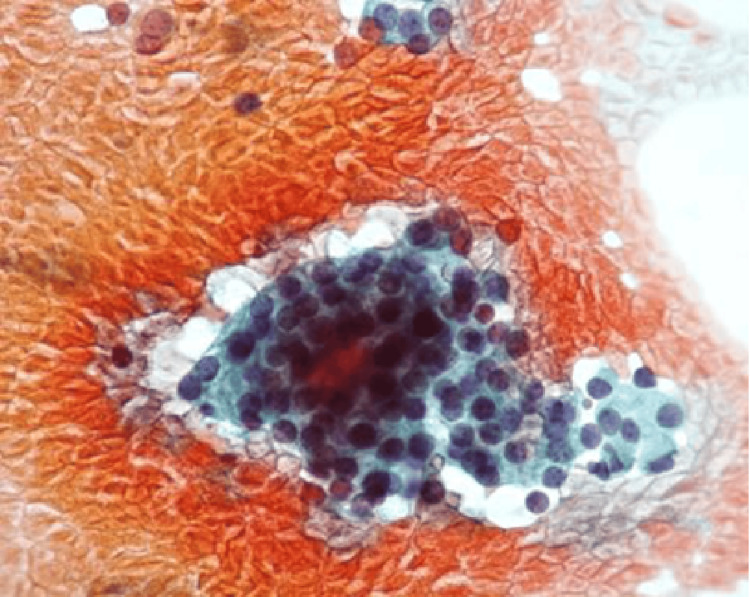
Parathyroid adenoma tissue section The tumor measured 35 x 25 mm. Histopathological examination confirmed the diagnosis of an adenoma, with no evidence of malignancy.

**Figure 4:**
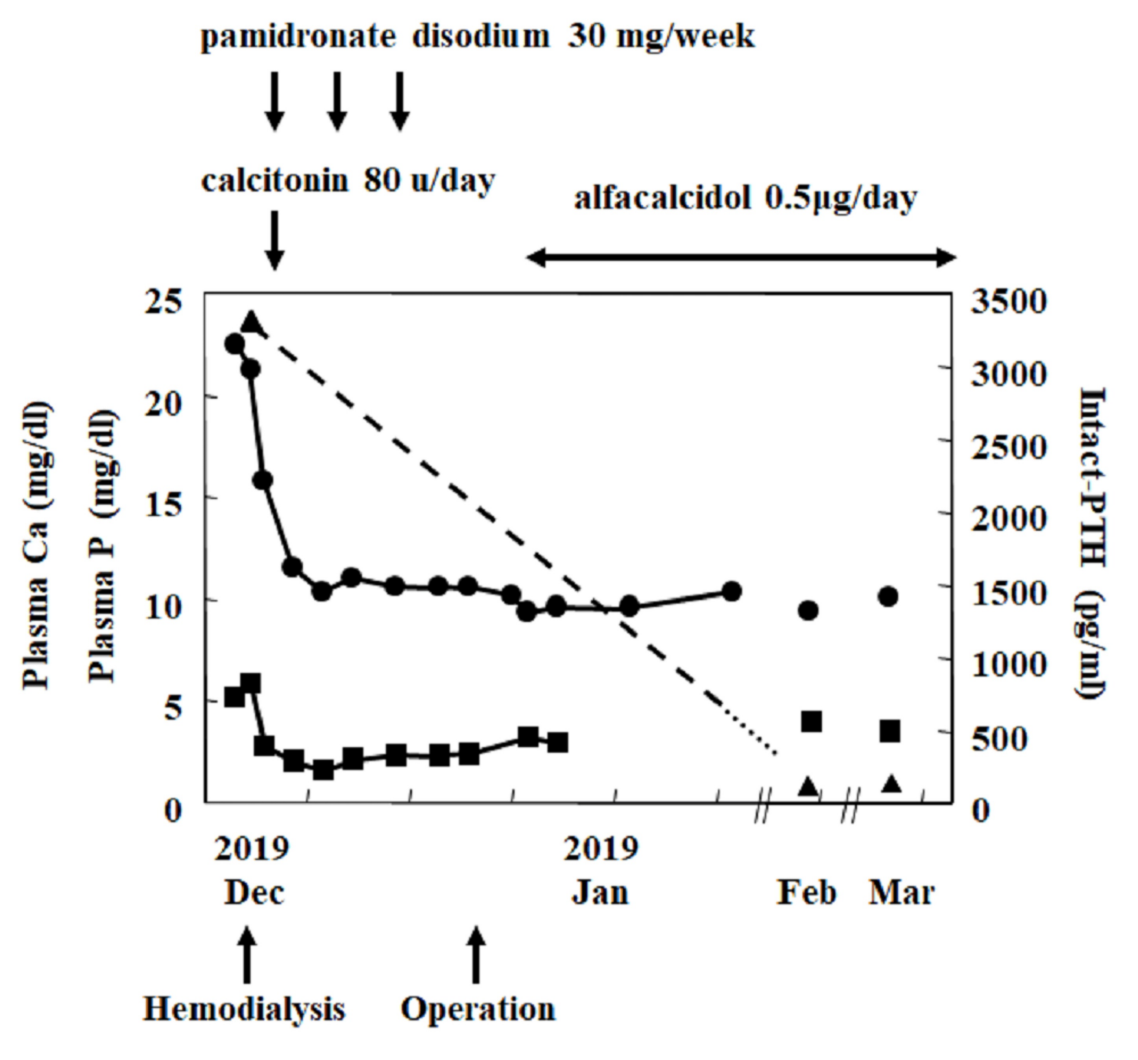
Clinical course of the patient The solid line with circles represents serum calcium levels. The solid line with squares represents serum inorganic phosphate levels. The dotted line with triangles represents serum intact PTH levels. PTH: parathyroid hormone

Eighteen days after admission, a parathyroidectomy was performed, targeting the left lower parathyroid gland. The resected gland was a substantial mass, measuring 35 x 25 mm, with no evidence of infiltration into the thyroid. Histological examination confirmed the diagnosis of parathyroid adenoma (Figure 3). The postoperative course was uneventful, with intact PTH levels gradually normalizing (96 pg/mL). By the day following surgery, serum calcium had normalized to 10.2 mg/dL (Figure 4). To prevent hypocalcemia, an active vitamin D3 analog (alfacalcidol 0.5 µg/day) was initiated. Follow-up showed stable calcium levels, and the patient remains asymptomatic. Six months after surgery, BMD improved at the distal radius (T-score: right; -2.48, left; -2.07) and showed a decreasing trend at the L2-4 spine (T-score: +1.18).

## Discussion

We report a case of PHPT presenting with a hypercalcemic crisis. Most hypercalcemia cases are caused by PHPT or malignancy but granulomatous disease, drug-induced hypercalcemia, and a few rare causes can also trigger this endocrine emergency [4,6-8]. In the past, PHPT was diagnosed only when patients presented with severe symptoms related to the effects of PTH on the kidneys and bones. However, with the advent of PTH assays, PHPT is now more easily diagnosed, and severe symptomatic hypercalcemia is becoming rare [4,5].

In this case of severe PHPT, bone minerals were markedly redistributed from cortical to trabecular bone. Generally, PHPT primarily affects cortical bone while trabecular bone tends to be relatively preserved. In patients with severe PHPT, osteosclerosis can also occur in areas with high trabecular bone content such as long canal diaphyses and vertebral bodies. It is thought that elevated PTH not only increases osteoclastic resorption of cortical bone but also promotes trabecular bone formation. This dual action of PTH on bone metabolism explains why high PTH concentrations stimulate bone resorption in cortical bone while also promoting osteoblastogenesis in trabecular bone [9]. In this case, the catabolic effect of PTH on cortical bone and its anabolic effect on trabecular bone occurred simultaneously, providing evidence that PTH can cause significant redistribution of bone minerals from cortical to trabecular bone. Recent studies using advanced imaging technologies, such as high-resolution peripheral computed tomography (HRpQCT) and trabecular bone structure assessment (TQCT), have revealed deterioration in trabecular microstructure in patients with PHPT [10,11]. Although trabecular bone may appear well-preserved in PHPT, bone strength could be compromised due to changes in microstructure.

Surgery was performed due to the severity of hypercalcemia. Within the first postoperative week, trabecular bone density increased while serum calcium, inorganic phosphate, and intact PTH levels normalized. Given the severity of the disease, the development of hungry bone syndrome postoperatively was expected [1], yet surprisingly, hypocalcemia was prevented with a relatively low dose of alfacalcidol. Considering the postoperative decrease in BMD at the L2-4 spine, it was thought that the supply of bone minerals from the trabecular bone may have contributed. Histopathological examination confirmed the diagnosis of a parathyroid adenoma, contrary to concerns about malignancy. However, the risk of recurrence in this patient must be carefully monitored.

## Conclusions

This case of a hypercalcemic crisis due to PHPT demonstrates an unusual pattern of bone mineral redistribution. The pronounced loss of cortical bone, combined with the preservation and enhancement of trabecular bone, underscores the complex skeletal effects of elevated PTH. Although hypercalcemic crises are rare, they remain clinically significant, requiring prompt intervention and long-term monitoring for potential recurrence or complications.

## References

[1] Walker MD, Silverberg SJ (2018). Primary hyperparathyroidism. Nat Rev Endocrinol.

[2] Griebeler ML, Kearns AE, Ryu E, Hathcock MA, Melton LJ 3rd, Wermers RA (2015). Secular trends in the incidence of primary hyperparathyroidism over five decades (1965-2010). Bone.

[3] Kim KJ, Baek S, Yu MH, Shin S, Cho S, Rhee Y, Hong N (2024). Secular trends in the incidence and treatment patterns of primary hyperparathyroidism in Korea: a nationwide cohort study. JBMR Plus.

[4] Walker MD, Shane E (2022). Hypercalcemia: a review. JAMA.

[5] Desmedt V, Desmedt S, D'heygere E, Vereecke G, Van Moerkercke W (2021). Hypercalcemia induced pancreatitis as a rare presentation of primary hyperparathyroidism. Acta Gastroenterol Belg.

[6] Jacobs TP, Bilezikian JP (2005). Clinical review: rare causes of hypercalcemia. J Clin Endocrinol Metab.

[7] Boonen S, Vanderschueren D, Pelemans W, Bouillon R (2004). Primary hyperparathyroidism: diagnosis and management in the older individual. Eur J Endocrinol.

[8] Stewart AF (2005). Clinical practice. Hypercalcemia associated with cancer. N Engl J Med.

[9] Rejnmark L, Ejlsmark-Svensson H (2020). Effects of PTH and PTH hypersecretion on bone: a clinical perspective. Curr Osteoporos Rep.

[10] Stein EM, Silva BC, Boutroy S (2013). Primary hyperparathyroidism is associated with abnormal cortical and trabecular microstructure and reduced bone stiffness in postmenopausal women. J Bone Miner Res.

[11] Hansen S, Beck Jensen JE, Rasmussen L, Hauge EM, Brixen K (2010). Effects on bone geometry, density, and microarchitecture in the distal radius but not the tibia in women with primary hyperparathyroidism: a case-control study using HR-pQCT. J Bone Miner Res.

